# Protective Effect of Penetratin Analogue-Tagged SOD1 on Cisplatin-Induced Nephrotoxicity through Inhibiting Oxidative Stress and JNK/p38 MAPK Signaling Pathway

**DOI:** 10.1155/2021/5526053

**Published:** 2021-08-21

**Authors:** Xiao-lu Wang, Liang Wang, Fo-lan Lin, Si-si Li, Ting-xuan Lin, Ren-wang Jiang

**Affiliations:** ^1^Guangdong Province Key Laboratory of Pharmacodynamic Constituents of TCM and New Drugs Research and International Cooperative Laboratory of Traditional Chinese Medicine Modernization and Innovative Drug Development of Chinese Ministry of Education, Jinan University, Guangzhou 510632, China; ^2^Department of Oncology, The First Affiliated Hospital of Jinan University, Guangzhou 510632, China; ^3^Shengshitaiyan (Guangdong) Health Tech Ltd., Nanhai District, Foshan 528231, China

## Abstract

Copper/zinc superoxide dismutase (SOD1) can clear cisplatin- (CP-) induced excessive reactive oxygen species (ROS), but exogenous SOD1 cannot enter cells because of its low biomembrane permeability. Cell-penetrating peptides (CPPs) can rapidly cross plasma membranes. This study is aimed at identifying an efficient and stable CPP-SOD1 and investigating its effects on CP-induced nephrotoxicity. We recombined SOD1 with 14 different CPPs and purified them using an NTA-Ni^2+^ column. In *in vitro* experiments, CPPs-SOD1 cell membrane penetration ability and JNK/p38 MAPK signaling pathway were evaluated using Western blotting. ROS production, mitochondrial membrane potential (MMP), and cell apoptosis were determined using flow cytometry and immunofluorescence staining in VERO and HK-2 cells. For *in vivo* experiments, mice were administered PSF-SOD1 for 2 h before cotreatment with a single CP injection for an additional 4 days. Blood and kidney samples were collected for renal function assessment (creatinine, urea nitrogen, histopathology, TUNEL assay, and JNK/p38 MAPK signaling pathway). Compared with TAT-SOD1, we found that PSF-SOD1 is more efficient at crossing the cell membrane and is stable after transduction into cells. Pretreatment with PSF-SOD1 inhibited CP-induced apoptosis, ROS generation, and JNK/p38 MAPK activation and restored CP-induced MMP loss in VERO and HK-2 kidney cells. Treatment of mice with PSF-SOD1 inhibited CP-induced serum creatinine, blood urea nitrogen elevation, and JNK/p38 MAPK activation. H&E staining and TUNEL assay indicated that kidney tissue damage was alleviated following PSF-SOD1 pretreatment. Overall, PSF-SOD1 ameliorated CP-induced renal damage by partially reducing oxidative stress and cell apoptosis by regulating JNK/p38 MAPK signaling pathway and might be a better cytoprotective agent than TAT-SOD1.

## 1. Introduction

Cisplatin (CP) is one of the most effective medications for the treatment of malignant diseases [1]; however, nephrotoxicity is still the major dose-limiting side effect and is observed in approximately 30% of patients [2]. Oxidative stress resulting from excessive production of reactive oxygen species (ROS) plays a critical role in CP-induced nephrotoxicity [3, 4]. When CP enters the cell, it reacts with intracellular antioxidants, disrupts the redox balance, and interferes with the respiratory chain in mitochondria, resulting in elevated ROS [5, 6]. Excess ROS can further consume intracellular antioxidants, damage mitochondria, and activate mitogen-activated protein kinases (MAPKs), including p38, *c*-Jun *N*-terminal kinase (JNK), and extracellular signal-regulated kinase 1/2 (ERK1/2), resulting in cell apoptosis [7–9]. Thus, clearing excess ROS and supplying endogenous antioxidants is important for ameliorating nephrotoxicity after CP administration.

To address this, antioxidant enzymes are a potent candidate. Copper/zinc superoxide dismutase (SOD1) is the most abundant intracellular antioxidant enzyme among SODs and can transform O2•^−^ to H_2_O [10]. However, the effect of exogenous SOD1 is limited due to its low cell membrane permeability. We could overcome this limitation by fusing SOD1 with cell-penetrating peptides (CPPs). Typically comprising 5–30 amino acids, this family of various peptides can cross plasma membranes rapidly [11]. CPPs-SOD1 is widely used for the treatment of ischemia, radiation, and inflammation-related diseases [12–14]. Among them, TAT-SOD1 has the most potent cell permeability, but it degrades within 3 h after transduction into cells [15]. Here, we selected 11 CPPs [16] to fuse with SOD1 and found a new recombinant protein, PEN-SOD1, whose cell permeability was comparable to that of TAT-SOD1. Penetratin (PEN) is an amphipathic CPP originating from the *antennapedia homeodomain* protein of Drosophila. Khafagy et al. found that PEN-modified amino acid sequences (PSF, PCR, and PCN) can enhance the absorption of insulin [17]. Therefore, we further fused PSF, PCR, and PCN with SOD1 and found that PSF-SOD1 was significantly more efficient and stable than TAT-SOD1. We assessed the protective activity of PSF-SOD1 in CP-induced nephrotoxicity models both *in vitro* and *in vivo*.

## 2. Materials and Methods

### 2.1. Construction of the CPPs-SOD1 Plasmid

CPPs-SOD1 expression vectors were constructed in our laboratory. CPPs-SOD1 genes were cloned from HK-2 cDNA using PCR. The sequences of the forward and reverse primers are listed in Table S1. Forward primers contain an NcoI restriction site, a sequence of CPPs, and PET-15b. Reverse primers contain an XhoI restriction site, sequences of six histidines, and PET-15b. Then, the CPPs-SOD1 gene was fused to NcoI-XhoI-digested pET15b by exnase (Vazyme, China). The sequencing results are shown in Figure S1.

### 2.2. Expression and Purification of CPPs-SOD1

CPPs-SOD1 plasmids were transformed into *E. coli* BL21 competent cells (Vazyme, China). The transformed cells were grown in LB medium at 37°C (OD_600_~0.5-1.0) and induced by 0.5 mM isopropyl-D-thiogalactoside (IPTG) (Aladdin, China) at 16°C for 16 h. Then, the bacterial cells were harvested and disrupted by sonication in PBS. After centrifugation, the supernatant crude enzyme was purified using Ni-NTA His-Tag Purification Agarose (MCE, USA). After washing with 10 volumes of equilibrium buffer (10 mM imidazole, 300 mM NaCl, 50 mM NaH_2_PO_4_, pH 8.0) and 10 volumes of clean buffer (50 mM imidazole, 300 mM NaCl, 50 mM NaH_2_PO_4_, pH 8.0), the fusion proteins were eluted with 10 volumes of elution buffer (300 mM imidazole, 300 mM NaCl, 50 mM NaH_2_PO_4_, pH 8.0). The salts in the protein fraction were removed using an ultrafiltration tube (Millipore, USA) and concentrated for subsequent experiments. The concentration and enzyme activity of purified CPPs-SOD1 were measured using a BCA protein assay kit (Vazyme, China) and SOD assay kit (Nanjing Jiancheng Bioengineering Institute, China), respectively.

### 2.3. Animals and Drug Treatments

Six- or seven-week-old male C57BL/6 mice were fed a standard laboratory diet and water *ad libitum*. Mouse care and animal handling were performed in accordance with the guidelines of the National Institutes of Health. The mice were divided into four groups: control, CP (15 mg/kg), CP+PSF-SOD1 (5 mg/kg), and CP+TAT-SOD1 (5 mg/kg). A mouse model of CP-induced renal injury was established by a single intraperitoneal injection of CP (15 mg/kg) [18, 19]. The indicated doses of PSF-SOD1 and TAT-SOD1 were administered intraperitoneally 2 h before CP injection and administered daily. The control group was administered saline alone. Four days later, all animals were sacrificed by cervical dislocation. Blood was collected for creatinine and urea nitrogen analysis; kidney samples were obtained for Western blot analysis and fixed in formalin for histological and TUNEL assay analysis.

### 2.4. Cell Culture

Green monkey kidney (VERO) cells and human proximal tubule epithelial cells (HK-2) were cultured in Dulbecco's modified Eagle's medium (Gibco, USA) supplemented with 10% fetal bovine serum (Gibco, USA), 100 units/mL penicillin, and streptomycin (Gibco, USA) in a humidified chamber at 37°C containing 5% CO_2_.

### 2.5. Cell Viability Assay

Cells were seeded into a 96-well plate at 1 × 10^4^ cells/well and treated with 1, 2, and 4 *μ*M PSF-SOD1, 4 *μ*M TAT-SOD1, and 4 *μ*M SOD1 for 1 h. Then, 10 *μ*M CP was added to each well and incubated for an additional 24 h. Cells were then treated with MTT solution (5 mg/mL) for 2 h, and the formazan was dissolved in 100 *μ*L dimethyl sulfoxide (DMSO). Absorbance was measured at 570 nm using a microplate reader (BioTek, USA).

### 2.6. Measurement of SOD Activity

After treatment with 1, 2, and 4 *μ*M PSF-SOD1, 4 *μ*M TAT-SOD1, and 4 *μ*M SOD1 for 1 h, or 4 *μ*M PSF-SOD1, 4 *μ*M TAT-SOD1, and 4 *μ*M SOD1 for 10, 30, and 60 min, VERO and HK-2 cells were lysed with PBS containing 0.5% Triton X-100. The lysate was then centrifuged at 12,000 rpm for 15 min. SOD activity in the supernatant was analyzed using SOD assay kits (Nanjing Jiancheng Bioengineering Institute, China) following the manufacturer's instructions.

### 2.7. Measurement of CPPs-SOD1 Cell Membrane Penetration Ability

VERO and HK-2 cells were seeded in 12-well plates and grown to 80% confluence. Then, 4 *μ*M CPPs-SOD1 was added to the medium for 1 h. Cells were then stained with SOD1 antibody for immunofluorescence analysis or lysed for Western blot analysis.

### 2.8. Measurement of PSF-SOD1 and TAT-SOD1 Stability

VERO and HK-2 cells were seeded in a 12-well plate and grown to 80% confluence. Then, 4 *μ*M PSF-SOD1 and 4 *μ*M TAT-SOD1 were added to the medium for 1 h. After removing the culture medium, cells were washed with PBS three times and replaced with fresh medium. After 1, 2, 4, 8, 12, and 24 h, the lysed cells were subjected to Western blot analysis to monitor the change in SOD1 levels.

### 2.9. Immunofluorescence

After treatment, cells were washed with PBS, fixed in 4% PFA for 20 min, and then washed and permeabilized with 0.5% TWEEN-20 for 20 min. After washing with PBS, cells were blocked with 10% goat serum for 1 h, then incubated with a primary specific antibody at 4°C overnight and FITC-conjugated secondary antibody for 2 h. The nuclei were counterstained with Hoechst stain. Fluorescence imaging was performed using an IN CELL Analyzer 6000 (GE Healthcare, USA).

### 2.10. Western Blot

After treatment, the cells were lysed with RIPA buffer and quantified. Equal volumes of protein were subjected to SDS-PAGE and transferred to polyvinylidene fluoride membranes. The membranes were then incubated with specific primary antibodies, washed, and incubated with HRP-conjugated secondary antibodies. Protein bands were detected using an enhanced chemiluminescence detection kit (Millipore, USA). The densitometry quantification of immunoblots was performed using ImageJ software.

### 2.11. Apoptosis Analysis

VERO cells were seeded in a 12-well plate and grown to 80% confluence. Then, 1, 2, and 4 *μ*M PSF-SOD1, 4 *μ*M TAT-SOD1, and 4 *μ*M SOD1 were added to the medium for 1 h before exposure to 10 *μ*M CP for an additional 24 h. The cell apoptosis rate was examined using the Annexin V-FITC/PI Apoptosis Detection Kit (Vazyme, China). Briefly, cells were collected, washed with PBS, suspended in Annexin-V binding buffer, and incubated with 5 *μ*L Annexin V-FITC and 5 *μ*L propidium iodide (PI) for 10 min. Samples were analyzed using a FACSCanto flow cytometer (BD, USA).

### 2.12. ROS Measurement

VERO cells were seeded in a 12-well plate and grown to 80% confluence. Then, 1, 2, and 4 *μ*M PSF-SOD1, 4 *μ*M TAT-SOD1, and 4 *μ*M SOD1 were added to the medium for 1 h before exposure to 1 mM CP for an additional 6 h. After treatment, cells were washed and incubated with 10 *μ*M DCFH-DA for 30 min at 37°C for immunofluorescence analysis using IN CELL Analyzer 6000 (GE Healthcare, USA) or harvested for quantitative assays with a FACSCanto flow cytometer (BD, USA).

### 2.13. Determination of Mitochondrial Membrane Potential (MMP)

VERO cells were seeded in a 12-well plate and grown to 80% confluence. Then, 1, 2, and 4 *μ*M PSF-SOD1, 4 *μ*M TAT-SOD1, and 4 *μ*M SOD1 were added to the medium 1 h before exposure to 1 mM CP for an additional 6 h. MMP was monitored using the JC-1 Mitochondrial Membrane Potential Detection Kit (Beyotime, China) according to the manufacturer's instructions. Briefly, cells were incubated with JC-1 solution for 30 min at 37°C and then washed three times with JC-1 buffer. When MMP decreased, the red fluorescence of JC-1 aggregates changed to green fluorescence of JC-1 monomers. The increase of green/red fluorescence intensity ratio presents the mitochondrial depolarization. The ratio of green/red fluorescence cells was quantified using a FACSCanto flow cytometer (BD, USA).

### 2.14. Histology

Mouse kidneys were fixed in 4% formalin, embedded in paraffin, sectioned (4 *μ*m), and stained with hematoxylin and eosin (H&E). Images were obtained using a Nikon Eclipse E800 microscope (Nikon, Japan).

### 2.15. TUNEL Assay

Apoptotic cells in kidney sections were detected using the One-Step TUNEL Apoptosis Assay Kit (Beyotime Biotechnology, China) according to the manufacturer's instructions. Briefly, the tissues were dehydrated, transparentized, impregnated, and embedded. Sections were then incubated with 3,3-diaminobenzidine (DAB), and the brown particles in the nucleus indicated positive staining.

### 2.16. Statistical Analysis

Data are expressed as mean ± SEM from at least three independent experiments. Data were analyzed using GraphPad Prism software (version 5.0). One-way ANOVA with Bonferroni multiple comparisons was used to analyze the differences between the groups. Statistical significance was set at *p* < 0.05.

## 3. Result

### 3.1. Screening for New Cell-Permeable CPP-SOD1

**Figure 1:**
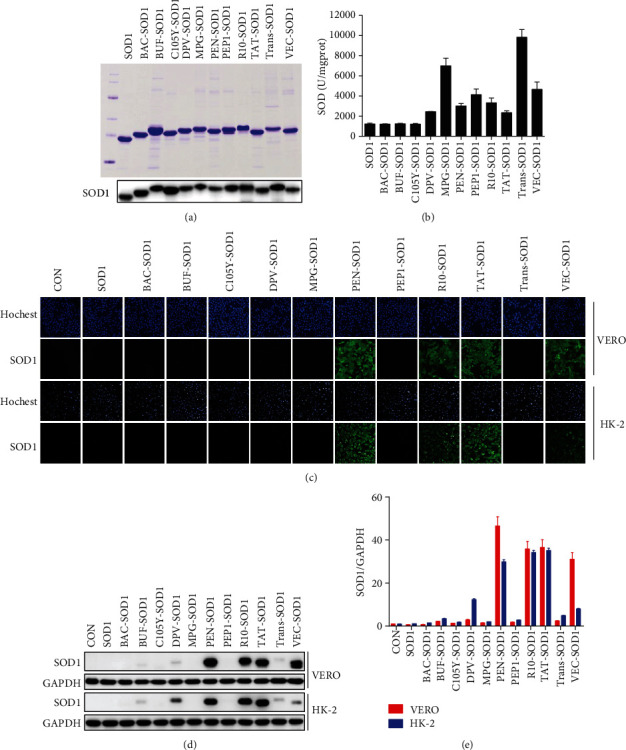
Screened PEN-SOD1 can efficiently penetrate the cell membrane. (a) 11 CPPs-SOD1 were expressed, purified, and detected by Coomassie brilliant blue staining (upper panel) and Western blot (lower panel). (b) SOD enzyme activity of 11 CPPs-SOD1 was measured according to the manufacturer's instructions. (c-e) VERO and HK-2 cells were treated with 4 *μ*M CPPs-SOD1 for 1 h. Cell membrane penetrating ability of 11 CPPs-SOD1 was determined by immunofluorescence staining with SOD1 (green) and Hoechst (blue) (c) and further analyzed by Western blot (d, e).

To find new cell-permeable CPP-SOD1, we selected 11 CPPs (Table 1) [16] to fuse with SOD1. After construction, expression, and purification, 11 CPPs-SOD1 were verified by Coomassie brilliant blue staining and Western blot analysis with SOD1 antibody (Figure 1(a)). The SOD activity of purified CPPs-SOD1 was approximately 3000 U/mg (Figure 1(b)).

Subsequently, we evaluated their cell-permeability abilities. Previous reports have shown that TAT and R_*n*_ have strong transduction properties [20]. Similarly, we found that TAT-SOD1 and R_10_-SOD1 could efficiently enter cells. Moreover, we found a new cell-permeable fusion protein, PEN-SOD1, which was comparable to TAT-SOD1 and R_10_-SOD1 using immunofluorescence analysis (Figure 1(c)) and Western blot analysis (Figures 1(d) and 1(e)). Since TAT-SOD1 has been widely studied for protection against oxidative stress-related diseases [14, 21], including CP-induced hepatocyte toxicity [22], we used TAT-SOD1 as a positive control.

### 3.2. PSF-SOD1 Has a Stronger Cell-Permeable Ability than TAT-SOD1

PEN is widely used as a highly efficient delivery carrier for poorly permeable therapeutic cargoes. As shown above, PEN-SOD1 is a new cell-permeable fusion protein, and its cell-permeable ability is comparable to that of TAT-SOD1. Khafagy et al. found that different PEN analogs influence the efficiency of insulin delivery [17]. Among them, PSF, PCR, and PCN (Table 1) increased insulin absorption compared to PEN. Thus, we fused the three analogs (PSF, PCR, and PCN) to SOD1, respectively, expecting to find a more efficient CPP-SOD1 than TAT-SOD1.

**Figure 2:**
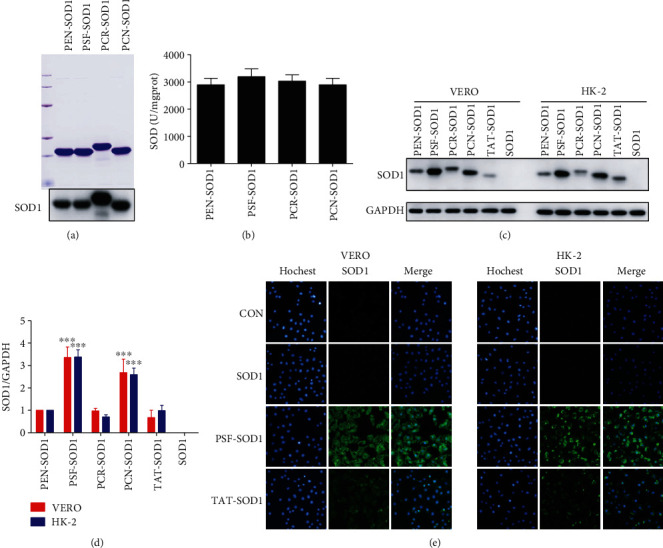
PSF-SOD1 has a higher cell-permeable ability than TAT-SOD1. (a) PEN analogs PSF, PCR, and PCN fused with SOD1 were expressed, purified, and detected by Coomassie brilliant blue staining (upper panel) and Western blot (lower panel). (b) SOD enzyme activity was measured according to the manufacturer's instructions. (c, d) Western blot analysis for cell permeability of fused SOD1 proteins. (e) Immunofluorescence analysis for cell permeability of fused SOD1 proteins with SOD1 antibody (green) and counterstained with Hoechst (blue). ^∗∗∗^*p* < 0.001 compared with TAT-SOD1-treated cells.

After construction, expression, and purification, PSF/PCR/PCN-SOD1 was verified by Coomassie brilliant blue staining and Western blot analysis with SOD1 antibody (Figure 2(a)). We also found that all of them showed SOD enzyme activity at approximately 3000 U/mg (Figure 2(b)). Next, we compared their cell-permeable ability with that of TAT-SOD1. The Western blot results showed that PSF-SOD1 had the most potent cell-permeable ability as compared to the others (Figures 2(c) and 2(d)). The immunofluorescence results further demonstrated that PSF-SOD1 has a stronger cell-permeable ability than TAT-SOD1 (Figure 2(e)). Therefore, we selected PSF-SOD1 for further investigations.

### 3.3. PSF-SOD1 Increases Intracellular Total SOD Enzyme Activity

**Figure 3:**
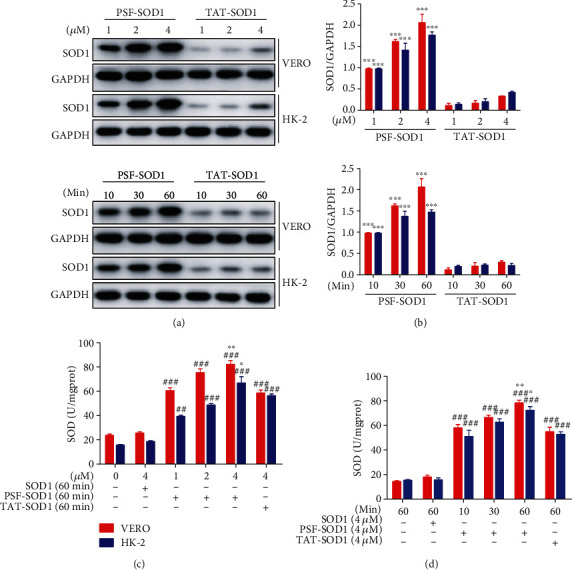
PSF-SOD1 increases intracellular total SOD enzyme activity. VERO and HK-2 cells were treated with 1, 2, 4 *μ*M PSF-SOD1 and 1, 2, 4 *μ*M TAT-SOD1 for 60 min or 4 *μ*M PSF-SOD1 and 4 *μ*M TAT-SOD1 for 10, 30, and 60 min. (a, b) Western blot analysis with SOD1 antibody in cell lysates. (c, d) Intracellular SOD enzyme activity in cell lysates was measured by SOD assay kit. ^##^*p* < 0.01, ^###^*p* < 0.001 compared with control cells. ^∗^*p* < 0.05, ^∗∗^*p* < 0.01, ^∗∗∗^*p* < 0.001 compared with TAT-SOD1-treated cells.

As PSF-SOD1 can rapidly cross the cell membrane, we examined whether it could elevate the total SOD activity in cells. We found that PSF-SOD1 and TAT-SOD1 can enter cells (Figures 3(a) and 3(b)) and increase the total SOD activity (Figures 3(c) and 3(d)) in a dose- and time-dependent manner. Moreover, PSF-SOD1 was significantly more efficient than TAT-SOD1 in penetrating the cell membrane and elevating intracellular SOD enzyme activity.

### 3.4. PSF-SOD1 Is Stable in VERO and HK-2 Cells

**Figure 4:**
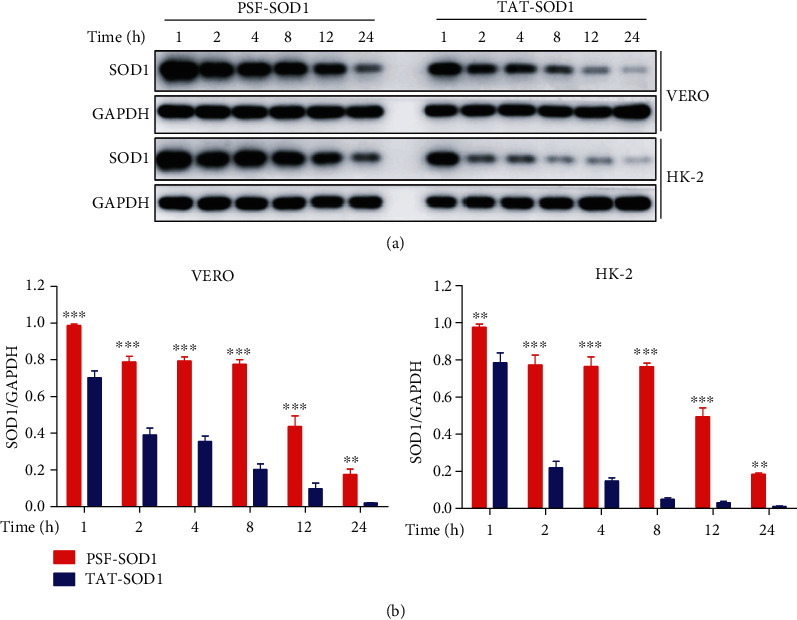
Intracellular PSF-SOD1 protein is more stable than TAT-SOD1. (a, b) VERO and HK-2 cells were treated with 4 *μ*M PSF-SOD1 or 4 *μ*M TAT-SOD1 for 1 h, and then, the cultured medium was removed and replaced with fresh medium. Cells were harvested at the indicated time points to analyze SOD1 protein level by Western blot. ^∗∗^*p* < 0.01 and ^∗∗∗^*p* < 0.001 compared with TAT-SOD1-treated cells.

After demonstrating that PSF-SOD1 was more efficient than TAT-SOD1 in cell transduction, we compared the stability of PSF-SOD1 and TAT-SOD1 after entering cells. It has been previously shown that TAT-SOD1 is rapidly degraded in 3 h [15]. In this study, we found that after 2 h, TAT-SOD1 degraded by half in VERO cells and degraded almost completely in HK-2 cells. In contrast, PSF-SOD1 remained almost unchanged until 24 h (Figures 4(a) and 4(b)). The stability of protein drugs in cells is crucial for therapeutic effects. Therefore, the significantly improved stability of PSF-SOD1 may contribute to its long-lasting effect on the antioxidant process.

### 3.5. PSF-SOD1 Reduces CP-Induced Apoptosis via the Mitochondrial Pathway

CPPs-SOD1 have been widely studied in various oxidative stress-related diseases [14, 23–25]; however, few studies have investigated their protection against toxicity induced by chemotherapeutic agents. Pan et al. found that the bifunctional antioxidant enzyme GST-TAT-SOD protected against CP-induced hepatocyte damage [22], but the protective effect of CPPs-SOD1 against CP-induced nephrotoxicity has not been studied previously. Therefore, we explored the effect of PSF-SOD1 on alleviating CP-induced renal injury.

**Figure 5:**
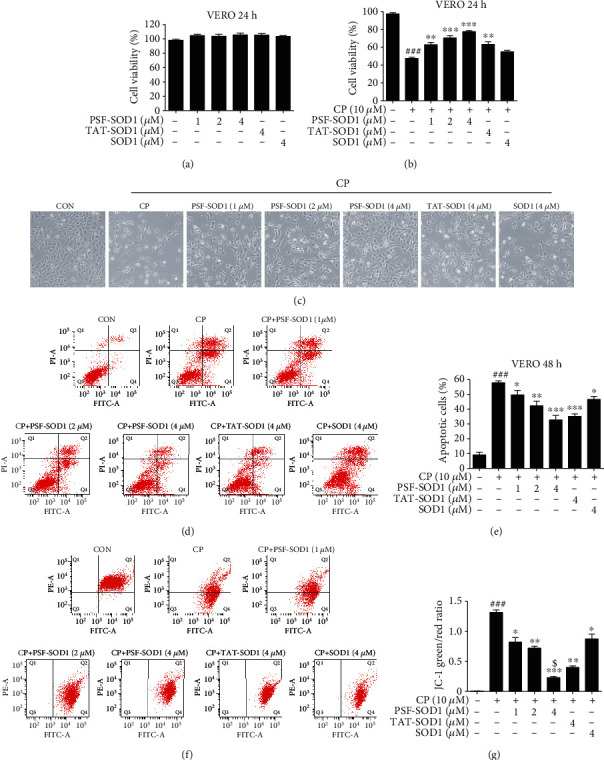
PSF-SOD1 inhibits CP-induced cell apoptosis by restoring MMP. VERO cells were treated with PSF-SOD1 and TAT-SOD1 with or without CP. (a, b) Cell viability was analyzed by MTT assay. (c) The morphological change was observed under a light microscope. (d, e) Cell apoptosis was tested by flow cytometry. (f, g) MMP levels were tested by flow cytometry. ^###^*p* < 0.001 compared with control cells. ^∗^*p* < 0.05, ^∗∗^*p* < 0.01, ^∗∗∗^*p* < 0.001 compared with the cells exposed to CP. ^$^*p* < 0.05 compared with TAT-SOD1-treated cells. MMP: mitochondrial membrane potential.

First, we investigated the toxicity of PSF-SOD1 and confirmed that a dose of 1-4 *μ*M was safe (Figure 5(a)). We found that PSF-SOD1 can alleviate CP-induced cell number decrease (Figure 5(b)), in accordance with the morphological changes observed under a light microscope (Figure 5(c)). Next, we examined whether PSF-SOD1 reduced CP-induced VERO cell apoptosis. The results showed that CP increased the apoptotic rate to approximately 60%, while PSF-SOD1 pretreatment lowered the apoptotic rate to 30% (for TAT-SOD1 was 40% and SOD1 was 50%) (Figures 5(d) and 5(e)).

Since cell apoptosis is involved in CP-induced mitochondrial dysfunction, which is accompanied by the loss of MMP [26–28], we next explored whether PSF-SOD1 could restore CP-induced MMP decrease. Control cells stained with JC-1 exhibited strong red fluorescence. CP treatment for 6 h significantly decreased the red/green fluorescence intensity ratio, indicating a loss of MMP. Meanwhile, PSF-SOD1 pretreatment reversed the CP-induced collapse of MMP, as indicated by an increased red/green fluorescence ratio. Moreover, PSF-SOD1 was more effective than TAT-SOD1 and SOD1 in restoring MMP (Figures 5(f) and 5(g)). The above results illustrated that PSF-SOD1 reduced CP-induced cell apoptosis by maintaining mitochondrial function.

### 3.6. PSF-SOD1 Reduces ROS Production and Inhibits the JNK/p38 MAPK Pathway

CP can increase intracellular ROS production and further activate MAPKs, including p38, JNK, and ERK1/2, resulting in cell apoptosis [29–31]. We investigated whether PSF-SOD1 could clear excess ROS and inhibit the activated JNK/p38 MAPK pathway induced by CP.

**Figure 6:**
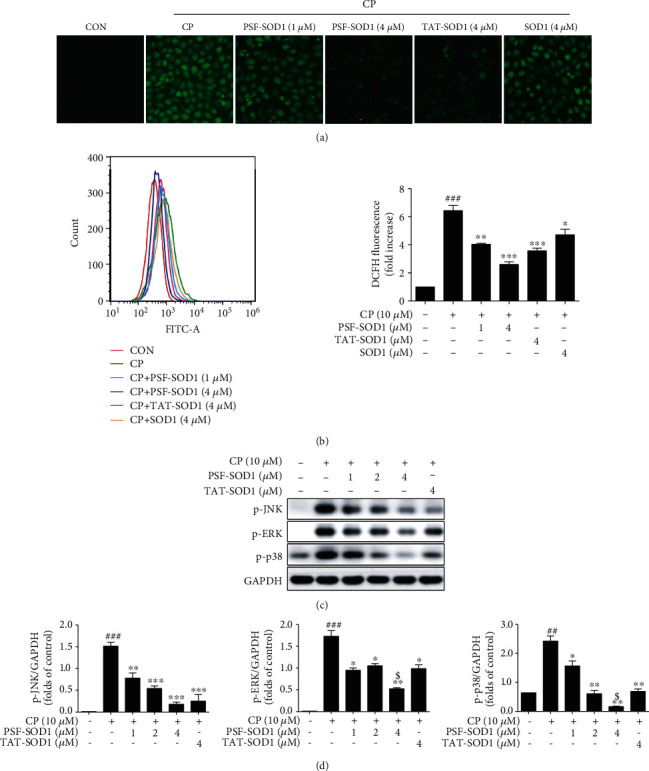
PSF-SOD1 inhibits CP-induced ROS production and JNK/p38 MAPK pathway activation. VERO cells were pretreated with PSF-SOD1, TAT-SOD1, and SOD1 for 1 h and then exposed to CP. (a) Images were acquired using a fluorescence microscope. (b) ROS-positive cells were quantified using flow cytometry. (c, d) Western blot analysis was performed using antibodies against p-JNK, p-ERK, and p-p38. ^##^*p* < 0.01 and ^###^*p* < 0.001 compared with control cells. ^∗^*p* < 0.05, ^∗∗^*p* < 0.01, ^∗∗∗^*p* < 0.001 compared with the cells exposed to CP. ^$^*p* < 0.05 compared with TAT-SOD1-treated cells.

The results showed that CP significantly increased intracellular ROS generation, and this effect could be reversed by PSF-SOD1 pretreatment (Figures 6(a) and 6(b)). Moreover, PSF-SOD1 had a stronger ROS-scavenging ability than TAT-SOD1 and SOD1. We also found that PSF-SOD1 inhibited the increase in the phosphorylation level of JNK/ERK/p38 induced by CP (Figures 6(c) and 6(d)), suggesting that PSF-SOD1 could alleviate CP-induced renal cell damage by scavenging ROS and inhibiting JNK/p38 MAPK pathway.

### 3.7. PSF-SOD1 Alleviates CP-Induced Renal Injury In Vivo

**Figure 7:**
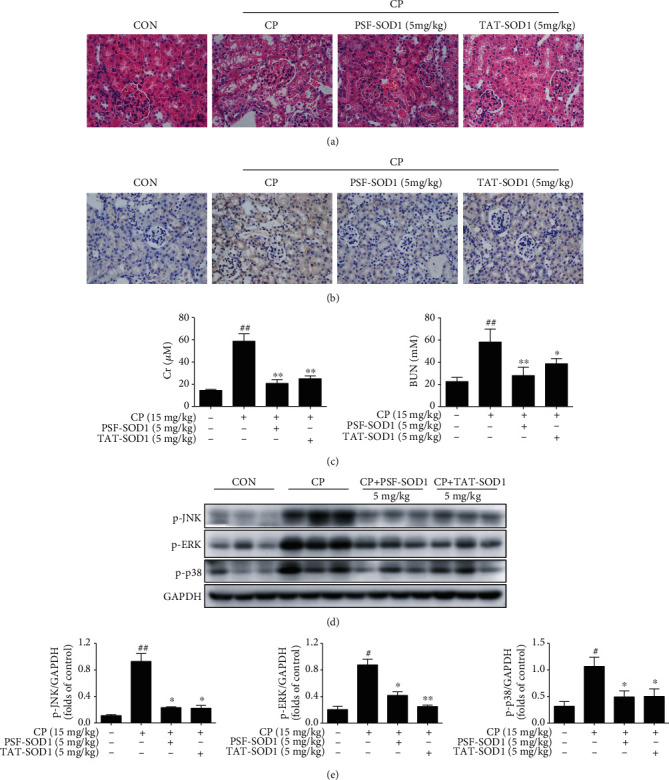
PSF-SOD1 alleviates CP-induced renal damage in vivo. (a) Representative histological photomicrograph of the kidney by H&E analysis. (b) TUNEL staining of the kidney. (c) Levels of serum creatinine (Cr) and blood urea nitrogen (BUN) were examined. (d, e) Western blot analysis of p-JNK, p-p38, and p-ERK in renal tissue lysates. ^##^*p* < 0.01 in comparison with control group. ^#^*p* < 0.05 and ^##^*p* < 0.01 compared with control cells. ^∗^*p* < 0.05 and ^∗∗^*p* < 0.01 compared with the CP-treated group.

Finally, we used a mouse model to further determine the therapeutic potential of PSF-SOD1. We found that pretreatment with PSF-SOD1 attenuated renal tubular epithelial cell vacuolization, necrosis, tubular atrophy (Figure 7(a)), and apoptosis (Figure 7(b)) and reduced the levels of creatinine and blood urea nitrogen (BUN) induced by CP (Figure 7(c)). In line with the *in vitro* results, PSF-SOD1 treatment inhibited CP-induced phosphorylation of JNK/ERK/p38 in mouse kidneys (Figures 7(d) and 7(e)). These results suggest that PSF-SOD1 could protect the kidneys from CP-induced damage *in vivo*.

## 4. Discussion

**Figure 8:**
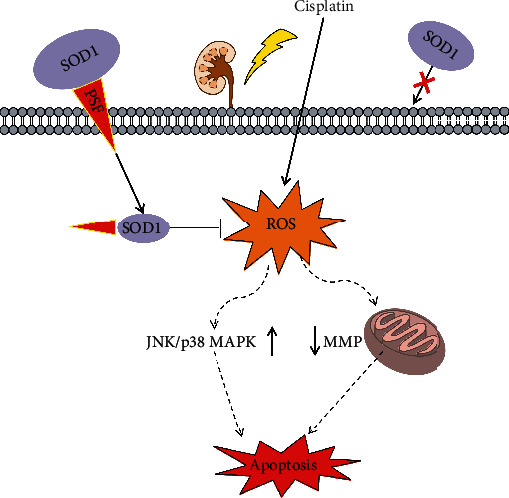
Schema of the potential antioxidant mechanism of PSF-SOD1.

Exogenous SOD1 is limited across the cell membrane because of low membrane permeability. We found a novel cell-permeable fusion protein, PSF-SOD1, which is more efficient and stable than TAT-SOD1, suggesting that PSF-SOD1 is more effective in the antioxidation process. TAT-SOD1 has been demonstrated to exert antioxidant effects in ischemia, radiation, and inflammation-related diseases [12–14], but little research has been conducted on its protective effect against CP-induced renal damage. In this study, we tested the potential therapeutic role of PSF-SOD1 and TAT-SOD1 in CP-induced nephrotoxicity *in vitro* and *in vivo*. The data showed that after entering the cells, PSF-SOD1 and TAT-SOD1 can clear CP-induced ROS, increase total SOD activity, restore MMP, inhibit JNK/p38 MAPK pathway, decrease cell apoptosis, and finally protect the kidneys against CP-induced injury (Figure 8). Moreover, the protective effect of PSF-SOD1 was more potent than that of TAT-SOD1. This finding suggests that PSF-SOD1 has potential applications in cancer therapy with CP to alleviate nephrotoxicity.

CP accumulates in the mitochondria, causes mitochondrial structural damage [26], increases ROS [32–35], activates the JNK/p38 MAPK pathway, and leads to apoptosis [9]. We found that PSF-SOD1 and TAT-SOD1 can clear excess ROS, restore MMP, and inhibit JNK/p38 MAPK activation induced by CP (Figures 5 and 6). Treatment with CP can decrease SOD activity [33], suggesting the role of SOD in CP-induced renal injury. SOD has been used in various oxidative stress-related diseases [36] with limited efficacy owing to its low cell penetrating ability. We can break the limitation by fusing SOD1 with CPPs, which can rapidly pass through the cell membrane rapidly.

CPPs are widely used in basic research, providing treatments for various diseases, such as diabetes, Parkinson's disease, amyotrophic lateral sclerosis (ALS), and Alzheimer's disease (AD) [37–40]. Many CPP-Cargos have entered phase II/III clinical trials, such as XG-102, which combines TAT with JNK inhibitors. A phase III clinical study was completed in 2016, confirming its ability to reduce intraocular inflammation and pain in patients undergoing cataract surgery [41, 42]. TAT-SOD1 can also exert antioxidant effects in ischemia, radiation, and inflammation-related diseases [12–14]. However, TAT-SOD1 is rapidly degraded after transduction into cells [15]. Consistently, we found that TAT-SOD1 degraded almost completely within 2 h after entering HK-2 cells, while PSF-SOD1 could still be detected in cells at 24 h (Figure 4). In addition, we found that PSF-SOD1 was more efficient than TAT-SOD1, suggesting that PSF-SOD1 has greater potential to sustain antioxidant effects.

Normally, the transduction ability of CPPs is related to arginine; for the guanidine, head group of arginine can form bidentate hydrogen bonds with the negatively charged carboxylic, sulfate, and phosphate groups of cell membrane components, leading to cellular internalization of CPPs [20]. TAT (RKKRRQRRR) and polyarginines (R_10_) fused with SOD1 have stronger transduction abilities compared with other CPPs-SOD1 (Figure 1). However, we found that the transduction efficiency of PEN-SOD1 (RQIKIWFQNRRMKWKK) with fewer arginine residues was similar to that of TAT-SOD1 (Figure 1). Furthermore, fixing Arg/Lys of PEN-SOD1 and reshuffling other amino acids [17] led to a penetration analog (PSF: RWFKIQMQIRRWKNKK). The new protein PSF-SOD1 showed approximately 10 times higher transduction efficiency than that of TAT-SOD1 (Figure 2). This result suggests that, except for Arg, the arrangement of other amino acids can also contribute to the penetrating properties of CPPs.

## 5. Conclusion

We found an efficient and stable cell-permeable fusion protein, PSF-SOD1, attenuated CP-induced nephrotoxicity through inhibition of ROS production, restoration of mitochondrial function, attenuation of JNK/p38 MAPK pathway activation, and cell apoptosis. Our results suggest that PSF-SOD1 has promising therapeutic potential for CP-mediated nephrotoxicity in cancer treatment.

## Figures and Tables

**Table 1:** Sequence of 14 cell-penetrating peptides (CPPs).

CPP name	Sequence
BAC	PRPLPFPRPG
BUF	RAGLQFPVGRVHRLLRK
C105Y	CSIPPEVKFNKPFVYLI
DPV1047	VKRGLKLRHVRPRVTRMDV
MPG	GALFLGFLGAAGSTMGAWSQPKKKRKV
PEN	RQIKIWFQNRRMKWKK
PEP1	KETWWETWWTEWSQPKKKRKV
R_10_	RRRRRRRRRR
TAT	RKKRRQRRR
Trans	GWTLNSAGYLLGKINLKALAALAKKIL
VEC	LLIILRRRIRKQAHAHSK
PSF	RWFKIQMQIRRWKNKK
PCR	RQIKIWFQNRRMKWKKRRRR
PCN	NRRMKWKKRQIKIWFQ

## Data Availability

All relevant data in the current study are available from the corresponding author (trwjiang@jnu.edu.cn) on request.

## Supplementary Materials

Supplementary Materials Table S1: PCR primer sequences listed for CPPs-SOD1. Figure S1: the sequencing results for the 14 CPPs-SOD1 (Supplementary Materials).
