# Supplementary material for: Protective Effect of Penetratin Analogue-Tagged SOD1 on Cisplatin-Induced Nephrotoxicity through Inhibiting Oxidative Stress and JNK/p38 MAPK Signaling Pathway
Source: Oxid Med Cell Longev. 2021 Aug 21;2021:5526053. doi: 10.1155/2021/5526053 (PMC8405295; doi:10.1155/2021/5526053)
Supplement: Supplementary Materials — Table S1: PCR primer sequences listed for CPPs-SOD1. Figure S1: the sequencing results for the 14 CPPs-SOD1 (Supplementary Materials). [file 5526053.f1.docx]

**Table S1.**

PCR primer sequences for CPPs-SOD1.

| CPP-SOD1 | Forward  primer (5’-3’) | Reverse primer (5’-3’) |
| --- | --- | --- |
| BAC-SOD1 | GAAGGAGATATACCATGCCGCGCCCACTGCCATTCCCTCGGCCGGGCGCGACGAAGGCCGTGTGCGTG | AGCAGCCGGATCCTCGAGTTAATGATGATGATGATGATGTTGGGCGATCCCAATTAC |
| BUF-SOD1 | GAAGGAGATATACCATGCGCGCAGGCCTGCAGTTTCCGGTTGGTCGCGTTCATCGCCTGCTGCGCAAAGCGACGAAGGCCGTGTGCGTG |  |
| C105Y-SOD1 | GAAGGAGATATACCATGTGTAGCATCCCGCCAGAAGTGAAATTCAACAAGCCGTTCGTGTATCTGATCGCGACGAAGGCCGTGTGCGTG |  |
| DPV1047-SOD1 | GAAGGAGATATACCATGGTTAAACGTGGACTTAAACTGCGGCACGTCAGACCGCGGGTGACGCGGATGGACGTCGCGACGAAGGCCGTGTGCGTG |  |
| MPG-SOD1 | GAAGGAGATATACCATGGGAGCCCTCTTCCTTGGCTTCCTTGGCGCCGCAGGCAGCACCATGGGTGCATGGAGCCAGCCGAAAAAGAAGCGCAAGGTGGCGACGAAGGCCGTGTGCGTG |  |
| PEN-SOD1 | GAAGGAGATATACCATGAGGCAAATAAAGATATGGTTCCAAAACAGACGAATGAAGTGGAAGAAGGCGACGAAGGCCGTGTGCGTG |  |
| PEP-1-SOD1 | GAAGGAGATATACCATGAAAGAAACCTGGTGGGAAACCTGGTGGACCGAATGGTCTCAGCCGAAAAAAAAACGTAAAGTGGCGACGAAGGCCGTGTGCGTG |  |
| R_10_-SOD1 | GAAGGAGATATACCATGCGTCGTCGTCGTCGTCGTCGTCGTCGTCGTGCGACGAAGGCCGTGTGCGTG |  |
| TAT-SOD1 | GAAGGAGATATACCATGAGGAAGAAGCGGAGACAGCGACGAAGAGCGACGAAGGCCGTGTGCGTG |  |
| Trans-SOD1 | GAAGGAGATATACCATGGGCTGGACCCTGAACAGCGCAGGCTACCTGCTCGGCAAGATCAACCTGAAGGCACTGGCAGCCCTTGCCAAGAAAATCCTGGCGACGAAGGCCGTGTGCGTG |  |
| VEC-SOD1 | GAAGGAGATATACCATGCTGCTGATCATTCTGCGCCGGCGCATCCGGAAACAGGCACACGCCCATAGCAAGGCGACGAAGGCCGTGTGCGTG |  |
| PSF-SOD1 | GAAGGAGATATACCATGAGGTGGTTCAAGATACAAATGCAAATAAGACGATGGAAGAACAAGAAGGCGACGAAGGCCGTGTGCGTG |  |
| PCR-SOD1 | GAAGGAGATATACCATGAGGCAAATAAAGATATGGTTCCAAAACAGACGAATGAAGTGGAAGAAGCGTCGGCGTCGGGCGACGAAGGCCGTGTGCGTG |  |
| PCN-SOD1 | GAAGGAGATATACCATGAACAGACGAATGAAGTGGAAGAAGAGGCAAATAAAGATATGGTTCCAAGCGACGAAGGCCGTGTGCGTG |  |

Trans-SOD1

CPP

SOD1


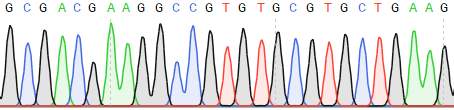

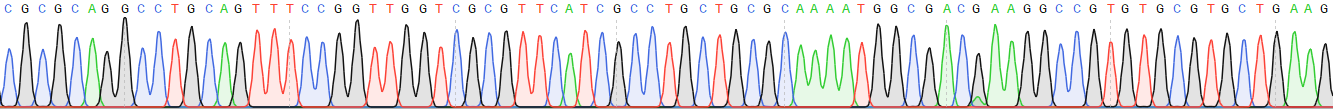

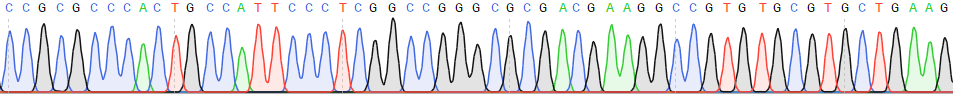

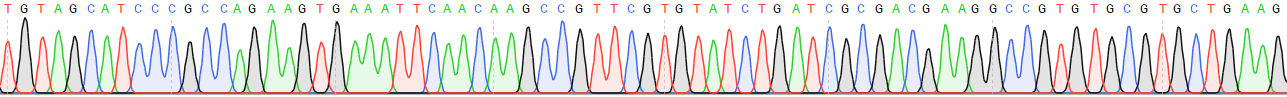

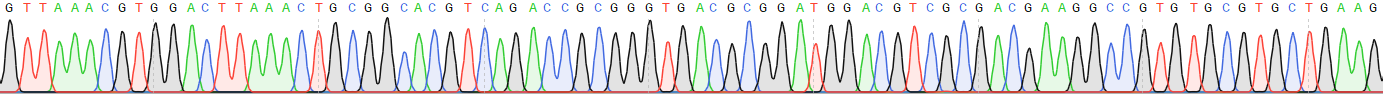

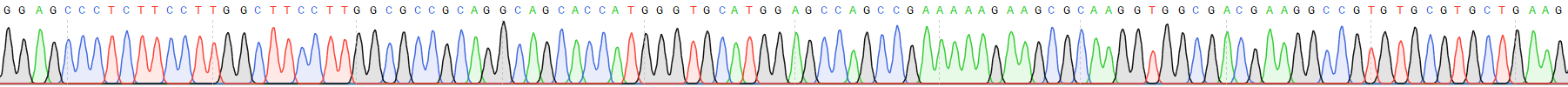

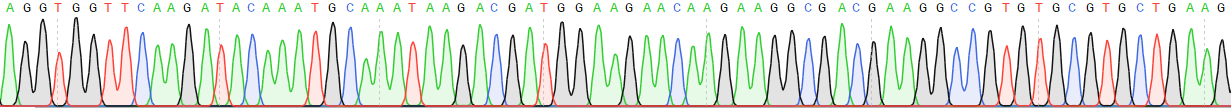

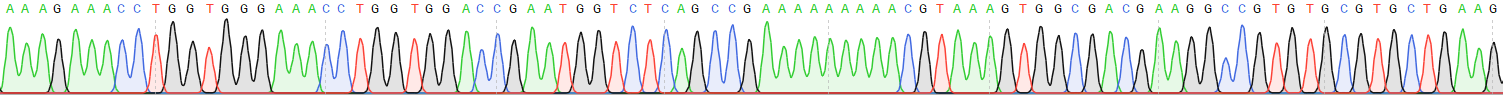

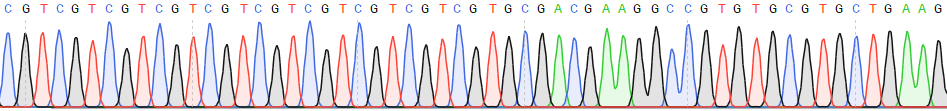

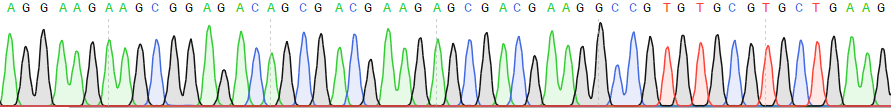

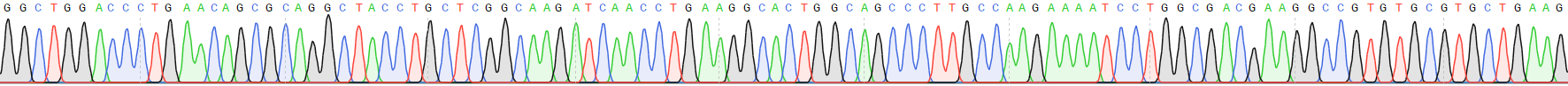

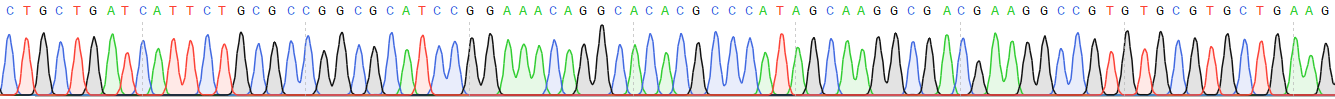

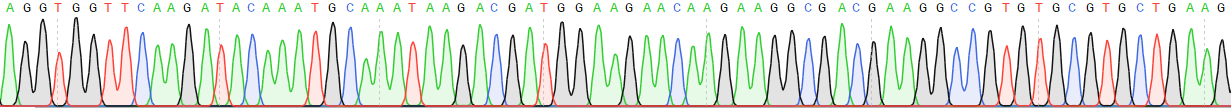

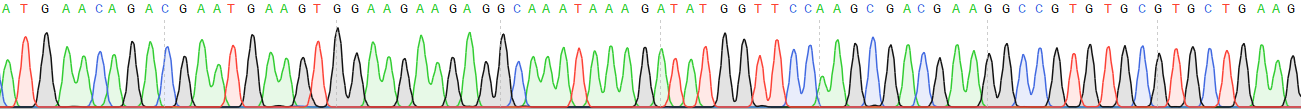

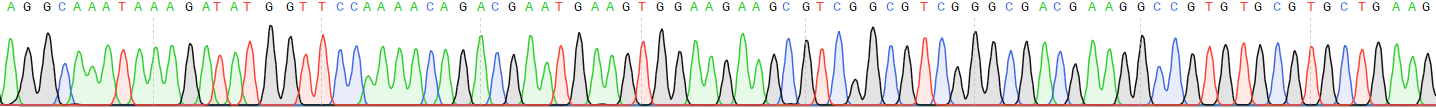


SOD1

BAC-SOD1

BUF-SOD1

C105Y-SOD1

DPV-SOD1

MPG-SOD1

PEN-SOD1

PEP-1-SOD1

R10-SOD1

TAT-SOD1

VEC-SOD1

PSF-SOD1

PCR-SOD1

PCN-SOD1

**Figure S1.** The sequencing results for the 14 CPPs-SOD1. We provided the whole CPP sequence and part of the SOD1sequence.
